# ERCC1 is a potential biomarker for predicting prognosis, immunotherapy, chemotherapy efficacy, and expression validation in HER2 over-expressing breast cancer

**DOI:** 10.3389/fonc.2022.955719

**Published:** 2022-10-20

**Authors:** Yilun Li, Xiaomei Liao, Li Ma

**Affiliations:** Fourth Hospital of Hebei Medical University, Shijiazhuang, China

**Keywords:** breast cancer, excision repair cross-complementary gene 1, human epidermal growth factor receptor-2, prognosis, clinicopathological feature

## Abstract

**Objective:**

To investigate the relationship between Excision repair cross-complementation 1 (ERCC1) expression, clinicopathological features, and breast cancer prognosis in patients treated with trastuzumab. Further, we aim to explore the immune status of ERCC1 in breast cancer.

**Methods:**

The data were retrieved from publicly available databases like the Cancer Genome Atlas, Therapeutically Applicable Research to Generate Effective Treatments, and the Genotype-Tissue Expression. The data was used to perform differential expression analyses between tumor and normal tissues in pan-cancers, immune-related analysis, homologous recombination deficiency (HRD), tumor mutation burden, and microsatellite instability. A total of 210 patients with HER2 over-expressing breast cancer from the Fourth Hospital of Hebei Medical University between January 2013 to December 2015 were enrolled in the study. Ten adjacent normal tissues were used to study the expression pattern of ERCC1 in normal tissues. Immunohistochemistry was performed to study ERCC1 expression and immune cell infiltration in different status of ERCC1 expression. Further, the correlation between ERCC1 expression, immune cell infiltration clinicopathological features, and the prognosis of patients with breast cancer was analyzed.

**Results:**

The immune analysis revealed a significant correlation between CD8+ T cell, CD4+ T cell, T helper cell, macrophages, mast cells, and ERCC1 expression. Spearman analysis show that ERCC1 expression is related to macrophages and T cells. A close correlation was observed between increased ERCC1 expression and high tumor immune dysfunction and exclusion (TIDE) score as well as HRD. The results revealed a significant correlation among ERCC1, chemotherapy and estrogen receptor (ER; P < 0.05) expression. Univariate survival analysis revealed a significant correlation (P < 0.05) between that ERCC1 and ER expression, blood vessel invasion, and disease-free survival (DFS). ERCC1 and ER expression, tumor size, blood vessel invasion, pathological type, and lymph node metastases significantly correlated (P < 0.05) with overall survival in patients. Multivariate regression analysis revealed that ERCC1 expression and chemotherapy were independent factors that influence DFS. ERCC1 expression and vascular tumor thrombus were independent influencing factors that influence OS.

**Conclusion:**

A correlation was observed between high ERCC1 expression and poor patient prognosis. High ERCC1 expression also influences the efficacy of immunotherapy and chemotherapy.

## Introduction

Breast cancer is one of the most common cancers in women. According to the statistics, in 2021, breast cancer accounted for 30% of all malignancies affecting females. Breast cancer ranks first in the number of incidences and second in cancer-related mortality (1). A higher incidence rate indicates a physical and psychological threat to women’s health. With continuous advancements in tumor immunology, molecular biology, and other disciplines, significant progress has been made to improve our understanding of breast cancer. Studies have been conducted to understand the pathogenesis of breast cancer at the cellular and molecular levels. To facilitate the identification and treatment of breast cancer, the medical community has classified breast cancer into luminal A, luminal B, human epidermal growth factor receptor (HER2) positive, and triple-negative breast cancer. Immunohistochemical markers have been used to classify tumors and have clinical significance since they aid in identifying therapeutic strategies based on molecular typing (2).

Comprehensive therapeutic strategies for breast cancer treatment primarily include surgery, chemotherapy, precision radiotherapy, hormone therapy, and targeted biological therapy. The therapeutic strategies are primarily based on clinical, pathological, and molecular characteristics. Notably, gene expression and mutation alter mRNA and protein expression, thereby altering the prognosis of patients (3). Gene mutation and uncontrolled proliferation of breast epithelial cells are underlying factors associated with occurrences of breast cancer (4). Therefore, exploring genes associated with cell proliferation and prognosis can help to predict the prognosis of patients with breast cancer.

Nucleotide excision repair cross-complementing gene 1 (ERCC1) is an important gene that encodes for a DNA repair protein and acts in concert with nucleotide excision repair cross-complementing gene 4 (ERCC4) to participate in nucleotide excision repair (5). Therefore, investigating the role of ERCC1 expression in cancer has always been the focus of research. A study explored the expression pattern of ERCC1in 51 patients with non-small cell lung cancer undergoing surgery to understand the correlation between ERCC1 expression and patient survival (6). The results showed a significantly higher median survival of ERCC1*-*positive patients compared to ERCC1-negative patients (94.9 months vs. 35.5 months (6). Another study has shown that patients with ERCC1*-*negative advanced non-small-cell lung cancer had better progression-free survival and overall survival (OS, P = 0.030) compared to ERCC1*-*positive patients (P = 0.016; (7). Further, an association between ERCC1 expression and higher pathological complete response (PCR) was observed (8).

Various studies have explored the effect of ERCC1 expression on the prognosis of a patient with different molecular subtypes of breast cancer. A study showed that high expression of ERCC1 in triple-negative breast cancer was associated with poor patient prognosis (9). The status of HER2 levels affects the expression of markers associated with drug resistance in cancers. Further, a correlation between ERCC1 expression and chemoresistance in cancer also exists. A study showed a decrease in chemoresistance, and an increase in the survival time was observed in patients with HER2 overexpressing gastric cancer and low ERCC1 expression compared to the patients with high ERCC1 expression. Further, a significant improvement in trastuzumab efficacy was observed in patients with HER2-positive gastric cancer and low ERCC1mRNA levels (10). These results indicate that HER2 overexpression and ERCC1 levels could enhance the efficacy of chemotherapy drugs. Therefore, ERCC1 expression should be studied in patients with other HER2 overexpressing cancers. This could aid in creating treatment strategies and identifying appropriate drugs to provide accurate and personalized therapy, thereby improving patient prognosis. The correlation between ERCC1 expression, patient prognosis, and clinicopathological features is still unclear in patients with HER2-positive breast cancer. Therefore, it is necessary to explore the ERCC1expression pattern to determine the effect of ERCC1 expression on the clinicopathological features and prognosis of patients with HER2-positive breast cancer treated with trastuzumab.

In this study, we first analyze the ERCC1 expression and the prognosis of patients with pan-cancer. Next, we performed an immune analysis to understand the role of ERCC1 expression in tumor immunity. Further, we studied the correlation between ERCC1 expression and microsatellite instability (MSI), homologous recombination deficiency (HRD), and tumor mutational burden (TMB) in pan-cancer. Finally, tissue samples of patients with HER2 over-expressing breast cancer were collected to study the expression and prognosis of patients. The data regarding HER2 (3+), HER2 (2+), and fluorescence *in situ* hybridization (FISH) (+) of the patient samples between January 2013 to December 2015 were obtained from the Fourth Hospital of Hebei Medical University. The study on patients with primary breast cancer was performed using amplification by FISH. Based on the expression of ERCC1, the patients were divided into negative, low, and high expression groups to explore the correlation between ERCC1expression, clinicopathological features, and prognosis.

## Materials and methods

### Expression analysis in pan-cancers

A standardized universal cancer dataset was retrieved using the University of California Santa Cruz (UCSC) (https://xenabrowser.net/) from databases like the Cancer Genome Atlas (TCGA), Therapeutically Applicable Research to Generate Effective Treatments (TARGET), Genotype-Tissue Expression (GTEx; PANCAN, N=19131, G=60499). Further, the ERCC1 (ENSG00000012061) expression data for samples were extracted. The samples were further screened as normal solid tissue, normal tissue, primary solid tumor, primary tumor, primary blood-derived cancer-bone marrow, and blood samples of primary peripheral blood-derived cancer. In addition, the sample with the 0 expression level was filtered, and a log2(x+0.001) transformation for each expression value was performed. Finally, we eliminated cancer with less than three samples in single cancer. The expression data of 34 cancer types were obtained for subsequent analysis. The data was visualized using the “ggplot2” R package.

### Survival analysis in pan-cancers

The samples with 0 expression level and patient follow-up < 30 days were filtered, and log2(x+0.001) transformation for each expression value was performed. The cancer type < 10 samples in a dataset of single cancer type were excluded from the analysis. Finally, 39 and 32 cancer types were obtained for OS and disease-free survival (DFS) analyses, respectively. The “survival” R package was used to perform survival analysis.

### Immune analysis in pan-cancers

The expression data of ERCC1 (ENSG00000012061), 150 immune pathways-associated genes (41 genes associated with chemokine, 18 receptor genes, 21 MHC-related genes, 24 immune-inhibitor genes, and 46 immune-stimulator genes), and 60 checkpoint genes (24 inhibitory and 26 stimulatory genes) were extracted (11). Further, the expression data of marker genes in each sample were screened. The sample sources were as follows: primary solid tumor, primary tumor, primary blood-derived cancer-bone marrow, and primary peripheral blood-derived cancer. Additionally, blood and normal samples with 0 expression levels were filtered, and log2(x+0.001) transformation for each expression value was performed. Pearson correlation analysis was performed on ERCC1 (ENSG00000012061) and the immune pathways and immune checkpoint-associated marker genes.

Tumor immune dysfunction and exclusion (TIDE) algorithm was used to predict the potential immune checkpoint blockade (ICB) response. The analysis was performed using clinical information of 186 patients with breast cancer and RNA-sequencing expression (level 3) data retrieved from the TCGA dataset (https://portal.gdc.com). The results were visualized using the “ggplot2” and “ggpubr” R package. Besides, RNA-sequencing expression (level 3) data and corresponding clinical information for breast cancer were retrieved from the TCGA dataset (https://portal.gdc.com). The “ggstatsplot” R package was used to visualize the correlation between gene expression and immune scores. Spearman’s rank correlation test was used to study the correlation between quantitative variables without normal distribution. The immune cell infiltration score for all patients in each type of tumor was assessed based on gene expression. The Deconvo_Cell-type Identification by Estimating Relative Subsets of RNA Transcripts (CIBERSORT) method was used to calculate immune scores in pan-cancers. P < 0.05 was considered statistically significant (12).

### Analysis of HRD, MSI, and TMB in pan-cancers

The data on TMB was retrieved from the Simple Nucleotide Variation (level 4) dataset for all TCGA samples using MuTect2 software (13). The MSI and HRD of samples were obtained from previous studies (14, 15). The TMB, MSI, HRD, and gene expression data were integrated. In addition, samples with 0 expression level were filtered, and log2(x+0.001) transformation was performed for each expression value. Finally, the dataset with less than 3 samples in a single cancer were eliminated. Finally, the expression data of 37 cancer types were obtained for subsequent analysis.

### Patient selection and information collection

Patients with HER2-positive primary breast cancer undergoing treatment from January 2013 to December 2015 at the Fourth Hospital of Hebei Medical University were selected for the study. The patient information was collected. All patients were treated with trastuzumab for one year, and the follow-up was conducted until March 30, 2021.

### Inclusion and exclusion criteria

The patient inclusion criteria were as follows: age ≥ 18 years; breast cancer patients diagnosed by puncture or resection; patients treated with trastuzumab-assisted targeted therapy for one year; normal liver, kidney, heart, and other main organs functions; Karnofsky score (KS) physical strength score ≥ 80 points. The exclusion criteria were: diagnosis of carcinoma in situ; male breast cancer; the presence of malignant tumors at other sites; incomplete clinical, follow-up, and histopathology data; unable to obtain complete information; patients with distant metastasis.

### Information on clinicopathological characteristics, calculation of ERCC1 expression score, and immune cell infiltration

Based on previous studies (8, 9), the patients were divided into two groups: ≤ 45 years and > 45 years. The patients were divided into three groups based on tumor size: ≤ 2 cm, > 2.1, and ≤ 5 cm, > 5 cm. Based on the pathological types, the patients were divided into invasive ductal carcinoma (IDC) and non-invasive ductal carcinoma. Based on the presence of vascular tumor thrombus, the patients were divided into with and without vascular tumor thrombus group. Lymph nodes were categorized into 0, 1-3, 4-9, and over 10, according to the number of metastases. The expression of estrogen receptor (ER) and progesterone receptor (PR) were categorized into < 1%, 1-10%, and > 10%. according to the detection and interpretation criteria of the immunohistochemical method. The boundary value of high and low expression of cell proliferating nuclear antigen (Ki-67) could be different in different pathological laboratories. The patients were divided into three groups in this study: ≤ 14%, 15–30%, and > 30% based on Ki-67 expression.

The expression of ERCC1was analyzed in patients. The information on the clinicopathological features, like age, menstrual status, tumor size, pathological type, lymph node metastasis, and the expression of ER, PR, and Ki-67, were collected. The relationship between the ERCC1 expression and clinicopathological features was analyzed.

Based on the inclusion criteria, the patients were selected for the study. The paraffin-embedded sections of patients were obtained based on the immunohistochemical data provided by the pathology department of the Fourth Hospital of Hebei Medical University. Immunohistochemistry was performed on the paraffin-embedded sections of patients. The sections were reexamined and interpreted based on relevant interpretation standards of ERCC1. The correlation between ERCC1 expression, clinicopathological features, and prognosis of patients was analyzed.

The sections were analyzed, and the results were interpreted by two senior pathologists. The expression pattern of ERCC1in patient samples was interpreted based on the widely used immunohistochemical scoring standard as follows: 1. Staining intensity: 0 when the sections were colorless, 1: light yellow staining, 2: yellow staining, and 3: brown staining; 2. Number of positive cells: 0 when the number of positive cells were ≤ 10%, 1: 11% to 25% positive cells, 2: 26% to 50% positive cells, and 3: > 50% positive cells. The average value of the two scores was calculated. ERCC1expression was considered high if the average value was > 2 scores. If the average value was 1–2 points (including 1 and 2 points), the ERCC1expression was considered low, and an average value < 1 point was regarded as a negative expression.

The correlation between ERCC1 expression score and immune cell infiltration was analyzed. Based on the bioinformatic analysis, the infiltration of T cells (CD3) and macrophages (CD206) were calculated, and the results were represented as % positive cells. The results were calculated using the formula: % Positive cell = Detected positive cell/Total cells. Spearman’s rank correlation analysis was performed to evaluate the correlation between ERCC1 expression score and immune cell infiltration.

### Follow-up and prognostic indicators

Telephone conversations, medical records, and archives were used to obtain patient information. The patients included in the study based on the inclusion criteria were followed up. The follow-up time was defined as the time from breast cancer diagnosis to the last follow-up or death. Breast cancer events were defined as local recurrence, distant metastasis, or patient death. Computed tomography scan, Magnetic resonance imaging, or biopsy, including regional lymph node recurrence and distant metastasis, were performed to confirm recurrence and metastasis in patients with breast cancer. The general clinicopathological data of the patients, including age, menstrual status, tumor size, pathological type, lymph node metastasis, chemotherapy status, immunohistochemistry (IHC), and FISH results, were collected retrospectively.

To study the relationship between ERCC1 expression and the prognosis of patients with different clinicopathological features, survival analysis was performed and were divided based on the clinical information stratified by other clinical information. Log-rank tests were used to perform survival analysis, and the results were represented as survival curves.

The outcomes were measured as OS and DFS. OS was defined as death due to any cause from the date of a breast cancer diagnosis. DFS is defined as the time elapsed from the date of breast cancer diagnosis to the time of disease recurrence or death.

### Statistical analysis

**Figure 1:**
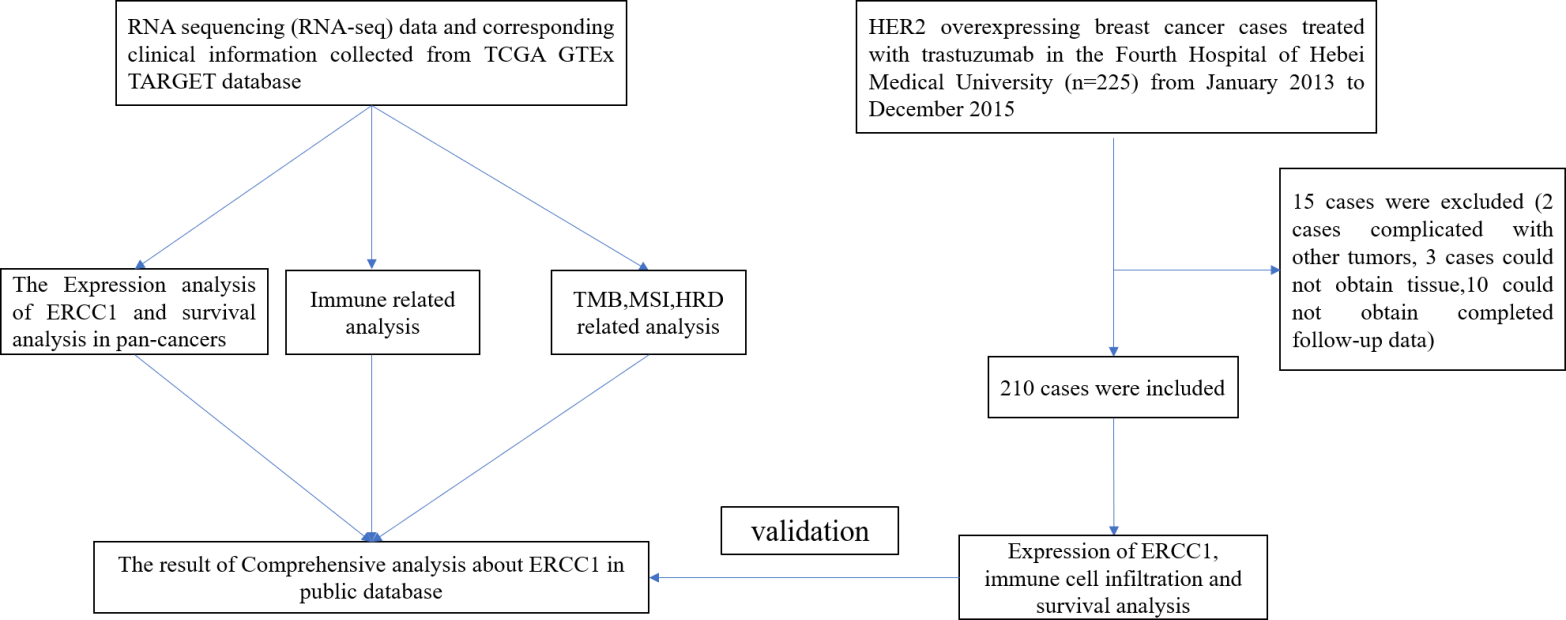
The flowchart of the complete data analysis and validation of ERCC1 expression.

SPSS 21.0 and R package 4.0.3 were used to analyze and tabulate the data. A chi-squared test was used to compare the clinicopathological characteristics of patients. The survival rate between groups was evaluated using Kaplan–Meier survival curve analysis. Multivariate regression analysis was performed to evaluate the effect of some factors on the patient prognosis using Cox proportional hazard model. P < 0.05 was considered statistically significant. The complete data analysis process is shown in **Figure 1**.

## Results

### Gene expression and patient survival analysis in pan-cancers

**Figure 2:**
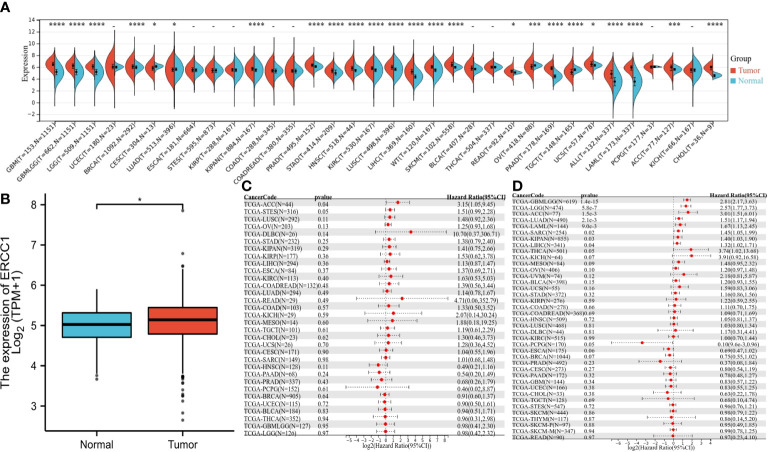
The expression of ERCC1 and survival analysis in pan-cancers, **(A)** ERCC1 expression in pan-cancers. **(B)** ERCC1 expression in breast cancer. **(C)** Univariate survival analysis in pan-cancers [disease-free survival (DFS]). **(D)** Univariate survival analysis in pan-cancers [overall survival (OS)]. *P < 0.05; ***P < 0.005; ****P < 0.001.

Unpaired Wilcoxon rank sum and Wilcoxon signed-rank test was used to performing analysis. The results revealed a significant increase in ERCC1 expression in 20 cancers, including GBMLGG, BRCA, HNSC, etc. Further, a significant decrease in ERCC1 expression was shown in patients with CESC, LUAD, OV, and TGCT (**Figure 2A**). The difference in ERCC1 expression between tumor cancer and normal tissue was analyzed, and the results revealed a significant increase in ERCC1 in breast cancer tumor tissue compared to normal tissue (**Figure 2B**).

The OS analysis revealed that in eight cancers (GBMLGG P = 1.4 e-15, LGG P = 5.8e-7, LUAD P = 2.1e-3, LAML P = 9.0e-3, SARC P=0.02, KIPAN P = 0.03, LIHC P = 0.04, ACC P = 1.5e-3) and low expression in PCPG (P = 0.05) had a poor prognosis (**Figure 2C**). The DFS analysis revealed that patients with high ERCC1 expression in ACC (P=0.04) had a poor prognosis (**Figure 2D**).

### HRD, MSI, TMB, and immune analysis in pan-cancers

**Figure 3:**
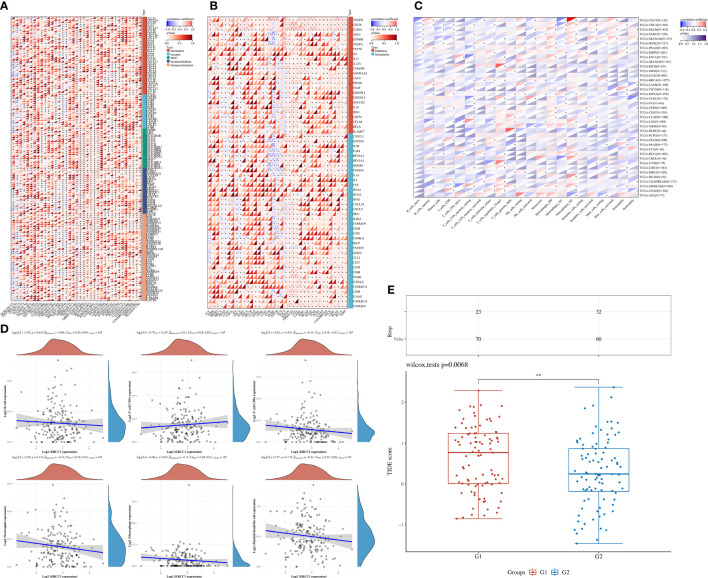
The effect of ERCC1 on immune status. **(A)** Correlation between ERCC1 and 150 immune pathways marker-related genes in pan-cancers. **(B)** Correlation between ERCC1 and immune checkpoints in pan-cancers. **(C)** The correlation between ERCC1 and tumor-associated immune cells was evaluated using Cell-type Identification by Estimating Relative Subsets of RNA Transcripts in pan-cancers. **(D)** Correlation between ERCC1 and tumor-associated immune cells in HER2 over-expressing breast cancer. **(E)** Tumor immune dysfunction and exclusion score in HER2 over-expressing breast cancer. G1: High expression of ERCC1. G2: Low expression of ERCC1 (Divided by average expression). *P < 0.05; **P < 0.01; ***P < 0.005; ****P < 0.001.

The aim of the pan-cancers analysis was to study the effect of ERCC1 expression on immune responses to identify patients with different types of cancers that may benefit from anti-ERCC1 immunotherapy. The results revealed a negative correlation between ERCC1 expression and most immunomodulators in patients with BRCA (**Figure 3A**). Furthermore, a negative correlation was observed between ERCC1 expression and most immune checkpoints (**Figure 3B**). However, no negative correlation was observed between immunomodulators or immune checkpoints and ERCC1 expression in patients with other cancers such as LGG, LAML, BLCA, and KIPAN. Further, we analyzed the relationship between ERCC1 expression and immune cell infiltration. As shown in **Figure 3C**, a significant correlation was observed between CD8^+^ T cells, CD4^+^ T cells, T helper cells, macrophages, mast cells, and ERCC1 expression. Further, the correlation between infiltrating immune cells and ERCC1 expression in patients with HER2 over-expressing breast cancer was evaluated. The results revealed a negative correlation between CD8^+^ T cell and ERCC1 expression (**Figure 3D**). Further, the TIDE score was calculated to evaluate the influence of ERCC1 on immunotherapy. The results revealed that in patients with HER2 over-expressing breast cancer, a positive correlation was observed between ERCC1 expression and TIDE score (**Figure 3E**), suggesting that ERCC1 expression could impact the effects of immunotherapy.

**Figure 4:**
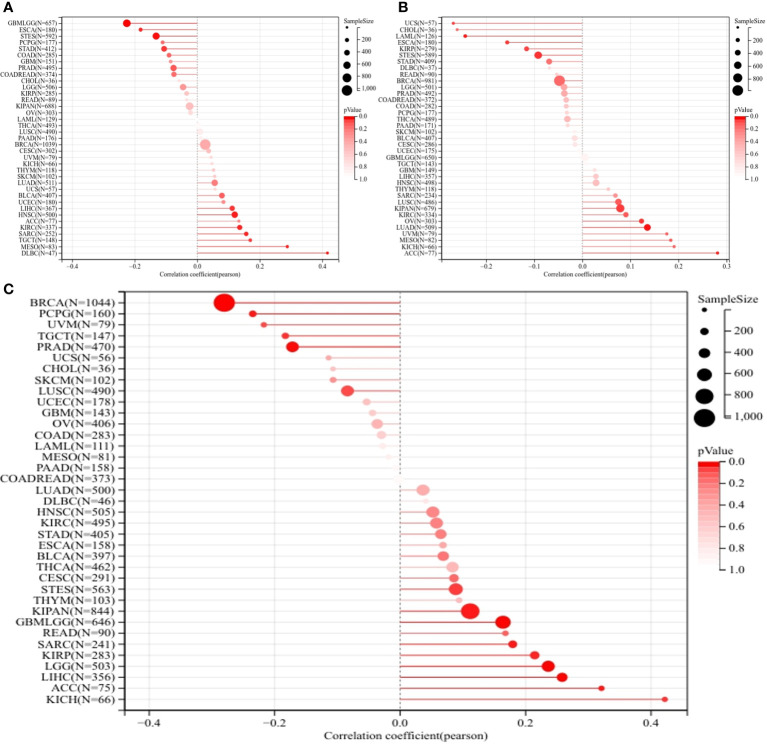
Homologous recombination deficiency (HRD), Microsatellite instability (MSI), and Tumor mutation burden (TMB) analysis in pan-cancers. **(A)** Correlation between ERCC1 and TMB in pan-cancers. **(B)** Correlation between ERCC1 and MSI in pan-cancers. **(C)** Correlation between ERCC1 and HRD in pan-cancers.

Further, no significant correlation was observed between ERCC1 expression and TMB and MSI in patients with breast cancer (**Figures 4A, B**). However, a negative correlation was observed between ERCC1 expression and HRD in patients with breast cancer (**Figure 4C**), thereby suggesting that ERCC1 expression may influence the efficacy of chemotherapy on patients with breast cancer.

### General information about patients

As shown in **Figure 1**, 225 patients preliminarily diagnosed with HER2-positive breast cancer were treated with trastuzumab between January 2013 to December 2015 Presence of other malignant tumors was observed in two patients with breast cancers. We could not obtain the paraffin-embedded tissue of three patients since the patients were diagnosed during surgical examination outside the hospital. Hence, these five cases were excluded from the analysis since they failed to meet the inclusion criteria. Therefore, 220 patients with HER2-positive breast cancer were included in the study. The follow-up of 10 patients could not be completed; therefore, the follow-up rate was 95.5%.

**Table 1** shows the general information and clinicopathological features of the patients. A total of 210 patients with HER2 (3+) breast cancer were enrolled (median age 49 years; range 27–73 years) for the study. Of the 210 patients with breast cancer, 82 (39%) were premenopausal, and 128 (61%) were menopausal. Based on the pathological types, 187 (87.6%) patients had IDC, no vascular tumor thrombus was observed in 147 (70%) patients, and no lymph node metastasis was observed in 123 (58.6%) patients. The status of immunohistochemical indicators in the patients was as follows: 108 (51.4%) patients with ER expression > 10%, 93 (44.3%) patients were PR negative, 132 (62.9%) patients had high Ki-67 expression, and 103 (49%) patients had low ERCC1 expression. Of the 210 patients followed up, recurrence and metastasis were observed in 28 patients (13.3%), 85.6% of patients had had five-year DFS, 22 patients (10.4%) died, and 88.3% of patients had five-year OS. The follow-up time was 4–98 months, and the median follow-up duration was 78 months.

**Table 1:** clinical baseline characteristics of 210 patients.

Characteristics		Value (IQR/proportion)
**Number of cases**		210
**Age of diagnosis (y), median (IQR)**		49 (40, 55)
**Menstrual status, n (%)**	Postmenopausal	82 (39%)
	Premenopausal	128 (61%)
**Tumor size (CM), n (%)**	<=2	79 (37.6%)
	2.1-5	124 (59%)
	>5	7 (3.3%)
**Intravascular cancer embolus, n (%)**	No	147 (70%)
	Yes	63 (30%)
**Pathologic classification, n (%)**	Invasive ductal carcinoma	184 (87.6%)
	Non-invasive ductal carcinoma	26 (12.4%)
**ER, n (%)**	<1%	78 (37.1%)
	1-10%	24 (11.4%)
	>10%	108 (51.4%)
**PR, n (%)**	<1%	93 (44.3%)
	1-10%	45 (21.4%)
	>10%	72 (34.3%)
**Ki-67, n (%)**	<=14%	22 (10.5%)
	15%-30%	56 (26.7%)
	>30%	132 (62.9%)
**ERCC1 expression, n(%)**	Negative	71 (33.9%)
	Low	103 (49.0%)
	High	36 (17.1%)
**Chemotherapy, n (%)**	Yes	136 (64.8%)
	No	74 (35.2%)
**Lymph node metastasis, n (%)**	0	123 (58.6%)
	1-3	46 (21.9%)
	4-9	24 (11.4%)
	>=10	17 (8.1%)

### Immunohistochemical staining of ERCC1 and immune cell infiltration in patients with different ERCC1 expression levels

**Figure 5:**
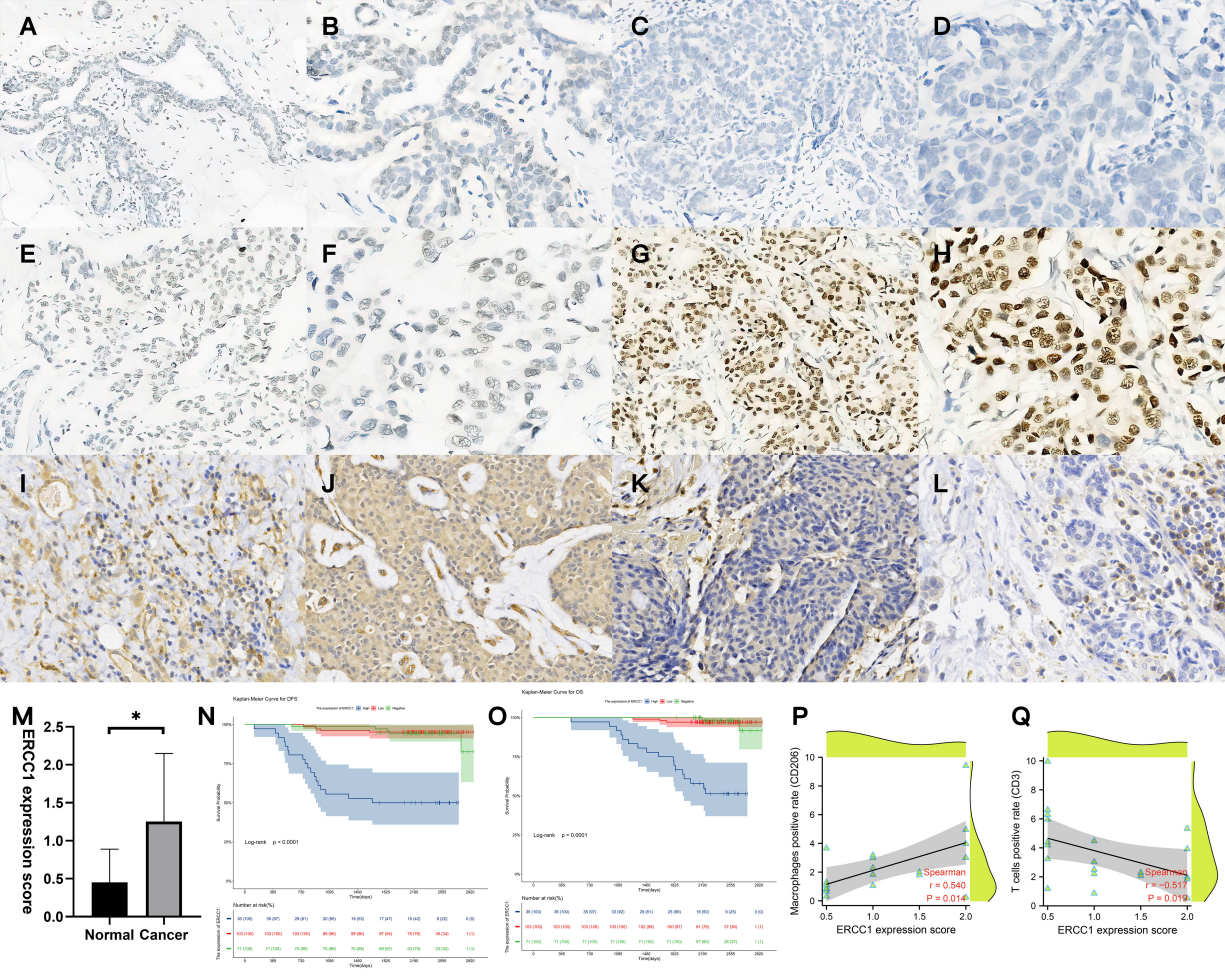
Validation of ERCC1 expression, survival analysis, and immune cell infiltration in patients. **(A, B)** Immunohistochemical (IHC) staining of ERCC1 in normal tissues, **(A)** Magnification, 10×10, **(B)** Magnification, 20×10. **(C, D)** IHC staining of ERCC1 in HER2 over-expressing breast cancer tissues (Expression: negative). **(C)** Magnification, 10×10, **(D)** Magnification, 20×10. **(E, F)** IHC staining of ERCC1 in HER2 over-expressing breast cancer tissues (Expression: low level). **(E)** Magnification, 10×10, **(F)** Magnification, 20×10. **(G, H)** IHC staining of ERCC1 in HER2 over-expressing breast cancer tissues (Expression: high level). **(G)** Magnification, 10×10, **(H)** Magnification, 20×10. **(I, J)** IHC localization of CD206 indicates macrophage infiltration in 20 patients with different levels of ERCC1 expression. **(I)** ERCC1 expression level: low, magnification, 20×10. **(J)** ERCC1 expression level: high, magnification, 20×10. **(K, L)** IHC localization of CD3 indicates T cell infiltration in 20 patients with different levels of ERCC1 expression. **(K)** ERCC1 expression level: low, magnification, 20×10. **(L)** ERCC1 expression level: high, magnification, 20×10. **(M)** Expression of ERCC1 in normal and cancer tissue. **(N)** Survival analysis grouped by ERCC1 expression (DFS), **(O)** Survival analysis grouped by ERCC1 expression (OS) Blue: high level, Red: low level, Green: negative. **(P)** Spearman’s rank correlation analysis determined the correlation between ERCC1 expression score and positive cell percentage of T cells (CD3). **(Q)** Spearman’s rank correlation analysis determined the correlation between ERCC1 expression score and positive cell percentage of macrophages (CD206). *P < 0.05.

A total of 210 HER2+ breast cancer and ten adjacent normal tissues were collected. The IHC results were interpreted based on the interpretation standard. The comprehensive score was based on the comprehensive staining intensity and the number of positive cells (**Figures 5A–H**). A significant increase in ERCC1 expression in breast cancer tissues compared to normal tissue (**Figure 5M**).

IHC performed to study immune cell infiltration revealed that patients with high expression of ERCC1 had significantly low T cell infiltration compared to patients with low expression of ERCC1. Further, significantly higher macrophage infiltration was observed in patients with high ERCC1 expression compared to patients with low ERCC1 expression (**Figures 5I–L**). Spearman’s rank correlation revealed a negative correlation between ERCC1 expression and infiltration of T cells, whereas a positive correlation between ERCC1 expression and macrophage infiltration (**Figures 5P, Q**).

### Relationship between ERCC1 expression and clinicopathological features

Chi-squared test was performed to study the correlation between ERCC1 expression and clinicopathological features. The results showed no significant correlation between ERCC1 expression and age at diagnosis, menopause, tumor size, vascular tumor thrombus, pathological type, PR and Ki-67 expression, and lymph node metastasis (*P* > 0.05). However, a significant (*P* < 0.05) correlation was observed between ERCC1and ER expression as well as chemotherapy (**Table 2**).

**Table 2:** Relationship between ERCC1 expression and clinical characteristics.

Characteristics	The expression level of ERCC1			P-value
	Negative	Low	High
**Number of cases**	71	103	36	
**Age of diagnosis, n (%)**				0.485
<=45	27 (12.9%)	42 (20%)	18 (8.6%)	
>45	44 (21%)	61 (29%)	18 (8.6%)	
**Menstrual status, n (%)**				0.327
Postmenopausal	32 (15.2%)	39 (18.6%)	11 (5.2%)	
Premenopausal	39 (18.6%)	64 (30.5%)	25 (11.9%)	
**Tumor size (CM), n (%)**				0.249
<=2	30 (14.3%)	38 (18.1%)	11 (5.2%)	
2.1-5	41 (19.5%)	60 (28.6%)	23 (11%)	
>5	0 (0%)	5 (2.4%)	2 (1%)	
**Intravascular cancer embolus, n (%)**				0.353
No	53 (25.2%)	72 (34.3%)	22 (10.5%)	
Yes	18 (8.6%)	31 (14.8%)	14 (6.7%)	
**Pathologic classification, n (%)**				0.426
Invasive ductal carcinoma	65 (31%)	89 (42.4%)	30 (14.3%)	
Non-invasive ductal carcinoma	6 (2.9%)	14 (6.7%)	6 (2.9%)	
**ER, n (%)**				**0.002**
<1%	21 (10%)	44 (21%)	13 (6.2%)	
1%-10%	3 (1.4%)	11 (5.2%)	10 (4.8%)	
>10%	47 (22.4%)	48 (22.9%)	13 (6.2%)	
**PR, n (%)**				0.061
<1%	23 (11%)	45 (21.4%)	15 (7.1%)	
1%-10%	16 (7.6%)	25 (11.9%)	14 (6.7%)	
>10%	32 (15.2%)	33 (15.7%)	7 (3.3%)	
**Ki-67, n (%)**				0.107
<=14%	7 (3.3%)	13 (6.2%)	2 (1%)	
15%-30%	27 (12.9%)	22 (10.5%)	8 (3.8%)	
>30%	37 (17.6%)	68 (32.4%)	26 (12.4%)	
**chemotherapy, n (%)**				
Yes	52 (24.8%)	68 (32.4%)	16 (7.6%)	**0.012**
No	19 (9.0%)	35 (16.7%)	20 (9.5%)	
**Lymph node metastasis, n (%)**				0.216
0	48 (22.9%)	55 (26.2%)	20 (9.5%)	
1-3	16 (7.6%)	23 (11%)	7 (3.3%)	
4-9	5 (2.4%)	15 (7.1%)	4 (1.9%)	
>=10	2 (1%)	10 (4.8%)	5 (2.4%)	

Bold values indicates P < 0.05.

### Survival analysis

Log-rank tests and univariate survival analyses were used to study the correlation between clinicopathological features, DFS, and OS in 210 patients with breast cancers. The results revealed no significant (*P* > 0.05) correlation between that patient’s age at diagnosis, menstrual status, tumor size, pathological type, PR, Ki-67, lymph node metastasis, and DFS. However, a significant correlation (*P* < 0.05) was observed between the ERCC1 and ER expression, vascular tumor thrombus, chemotherapy, and DFS (**Table 3**). No correlation was observed between the patient’s age at diagnosis, menstrual status, Ki-67 levels, PR expression, and OS (*P* > 0.05). However, a significant correlation (*P* < 0.05) was observed between ERCC1 expression, vascular tumor thrombus, pathological type, ER, tumor size, chemotherapy, lymph node metastasis, and OS (**Table 3**).

**Table 3:** Univariate analysis of DFS and OS in the collection of 210 patients.

Characteristics	Event			
	DFS, n(%)	*P-*value	OS, n(%)	*P-*value
**Number of cases**	28		22	
**ERCC1 expression, n(%)**		**< 0.001**		**< 0.001**
Negative	5 (2.4%)		2 (1%)	
Low	5 (2.4%)		3 (1.4%)	
High	18 (8.6%)		17 (8.1%)	
**Age of diagnosis, n (%)**		0.711		0.526
<=45	13 (6.2%)		11 (5.2%)	
>45	15 (7.1%)		11 (5.2%)	
**Menstrual status, n (%)**		0.153		0.059
Postmenopausal	7 (3.3%)		4 (1.9%)	
Premenopausal	21 (10%)		18 (8.6%)	
**Tumor size (CM), n (%)**		0.073		**0.029**
<=2	11 (5.2%)		9 (4.3%)	
2.1-5	14 (6.7%)		10 (4.8%)	
>5	3 (1.4%)		3 (1.4%)	
**Intravascular cancer embolus, n (%)**		**0.024**		**0.004**
No	14 (6.7%)		9 (4.3%)	
Yes	14 (6.7%)		13 (6.2%)	
**Pathologic classification, n (%)**		0.057		**0.037**
Invasive ductal carcinoma	21 (10%)		16 (7.6%)	
Non-invasive ductal carcinoma	7 (3.3%)		6 (2.9%)	
**ER, n (%)**		**0.012**		**0.028**
<1%	10 (4.8%)		9 (4.3%)	
1%-10%	8 (3.8%)		6 (2.9%)	
>10%	10 (4.8%)		7 (3.3%)	
**PR, n (%)**		0.462		0.718
<1%	10 (4.8%)		9 (4.3%)	
1%-10%	10 (4.8%)		7 (3.3%)	
>10%	8 (3.8%)		6 (2.9%)	
**Ki-67, n (%)**		0.954		0.428
<=14%	3 (1.4%)		4 (1.9%)	
15%-30%	8 (3.8%)		6 (2.9%)	
>30%	17 (8.1%)		12 (5.7%)	
**Chemotherapy**		**0.001**		**0.003**
Yes	10 (4.8%)		8 (3.8%)	
No	18 (8.6%)		14 (6.7%)	
**Lymph node metastasis, n (%)**		0.125		**0.039**
0	18 (8.6%)		13 (6.2%)	
1-3	2 (1%)		1 (0.5%)	
4-9	5 (2.4%)		5 (2.4%)	
>=10	3 (1.4%)		3 (1.4%)	

Bold values indicates P < 0.05.

**Figure 6:**
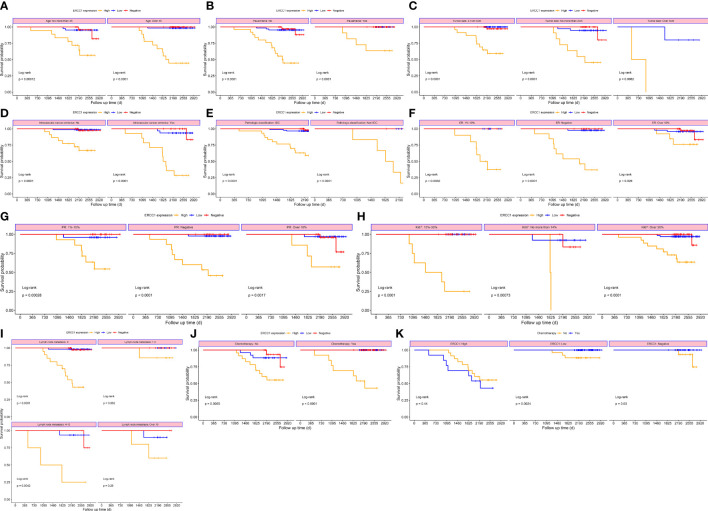
Survival curves stratified based on clinical factors (OS) **(A)** Age **(B)** menopause **(C)** Tumor size **(D)** Intravascular cancer embolus **(E)** Pathological classification **(F)** ER **(G)** PR **(H)** Ki-67 **(I)** Lymph node metastasis **(J)** Chemotherapy **(K)** ERCC1 expression.

**Figure 7:**
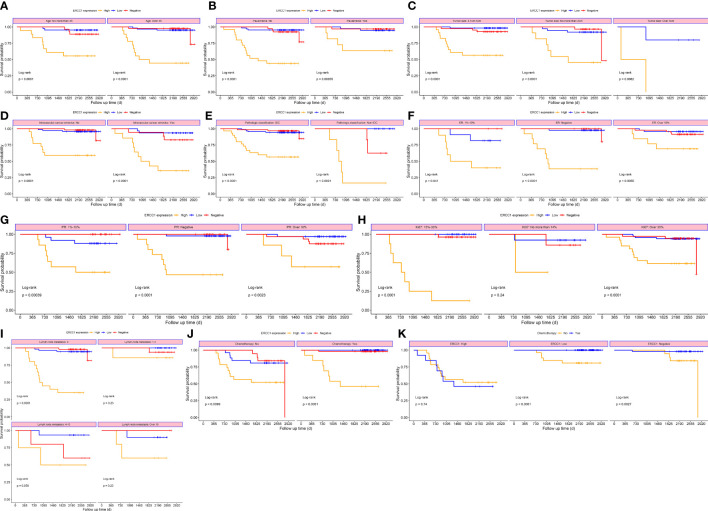
Survival curves stratified based on clinical factors (DFS) **(A)** Age **(B)** menopause **(C)** Tumor size **(D)** Intravascular cancer embolus **(E)** Pathologic classification **(F)** ER **(G)** PR **(H)** Ki-67 **(I)** Lymph node metastasis **(J)** Chemotherapy **(K)** ERCC1 expression.

To study the correlation between ERCC1 expression and patient prognosis in patients with different clinicopathological features, survival analysis was performed, stratified by clinicopathological features. The result revealed a significant correlation (*P* < 0.05) between OS and DFS stratified by all clinical factors (Age, menstrual status, Ki-67, ER and PR expression, vascular tumor thrombus, pathological type, tumor size, chemotherapy, and lymph node metastasis; **Figures 6**, **7**).

**Figure 8:**
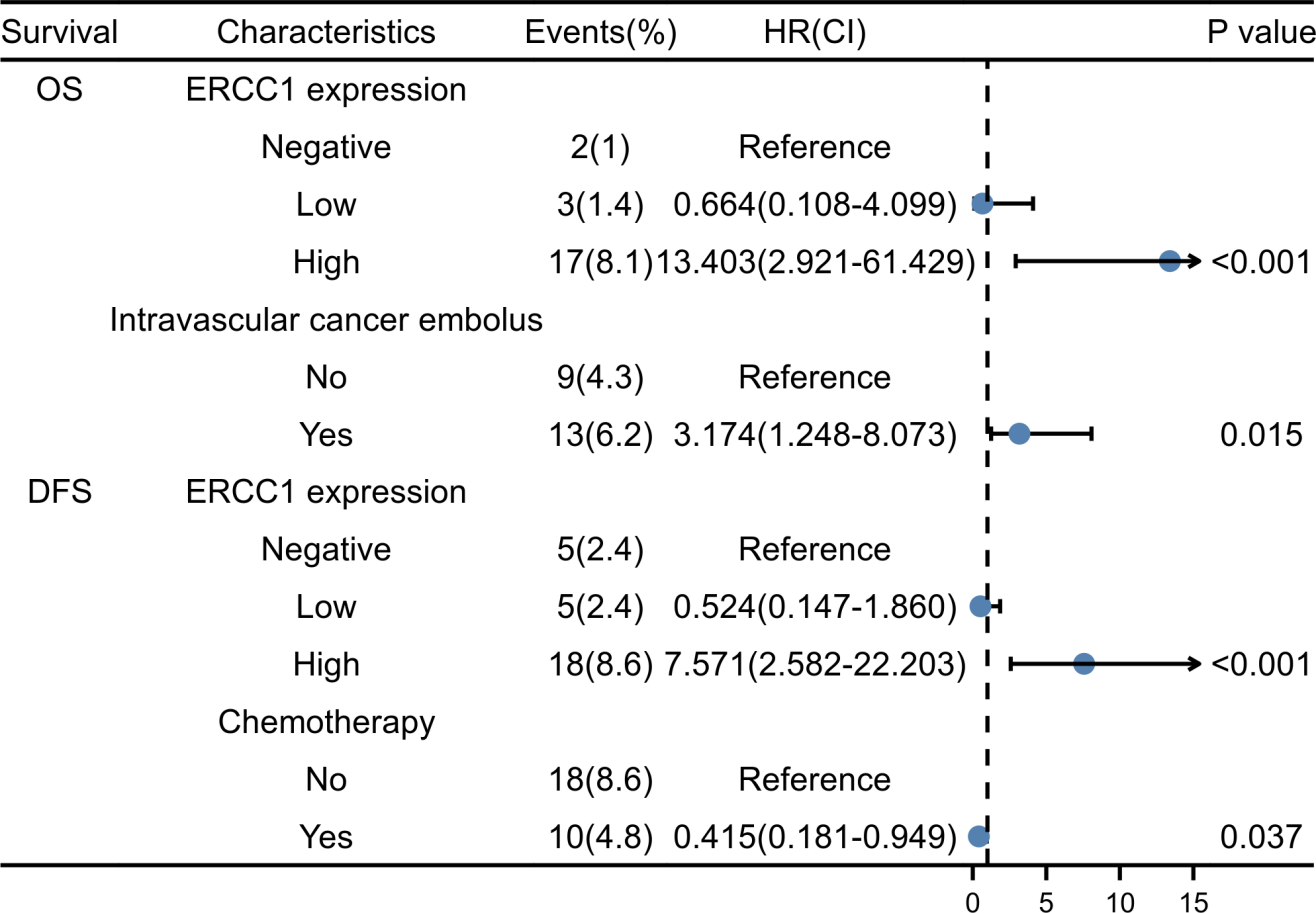
The forest plot of multivariate COX survival analysis.

Multivariate regression analysis was performed using Cox proportional hazards on parameters identified as significant indicators using the univariate analysis. As shown in **Figures 5N, O**, the Kaplan–Meier survival curve indicates the correlation between ERCC1 expression, DFS, and OS. The independent factors that influence the DFS and OS are shown in the forest map. The forest map shows that ERCC1 expression [P < 0.001, HR = 11.349 (4.108–31.355)] and chemotherapy [P <0.05, HR = 0.415(0.181–0.949)] were independent factors that influence DFS. ERCC1 expression [*P* <0.001, HR = 13.403 (2.921–61.429)] and vascular tumor thrombus [*P* < 0.05, HR = 3.174(1.248–8.073)] were independent influencing factors that influence OS (**Figure 8**).

## Discussion

Breast cancer is the most common cancer in women, and its incidences are gradually increasing. In 2021, 280000 new cases and 40000 deaths related to breast cancer were reported. Breast cancer has the highest incidence rate, surpassing lung cancer (1, 16).

The advancement in technology like DNA sequencing has allowed us to explore the mechanism underlying the occurrence, development, and metastasis of breast cancer. Further, it also allows us to explore the factors that could influence the prognosis of patients using clinical data. ER, PR, HER2, and Ki-67 are commonly used markers for classifying different breast cancer types using IHC. This aids in designing personalized treatment for patients with breast cancer. Based on the expression of IHC markers, breast cancers are classified as luminal A, luminal B, triple-negative, and HER2 overexpressing (17). HER2 overexpression usually refers to an IHC score of 3+ or 2+ and FISH amplification, accounting for approximately 25% of all breast cancer cases. High HER2 level activates AKT, which triggers anti-apoptotic signaling to enhance the TNF-α resistance phenotype of cancer cells. HER2 usually forms a homodimer with other HER2 proteins, which activates the Ras/Raf/MAPK and PI3K/Akt signaling pathway to promote the proliferation and metastasis of tumor cells (18). Trastuzumab is used for treating patients with HER2 overexpressing breast cancer. Trastuzumab increases tumor cell susceptibility by altering HER2 expression, thereby increasing the drug efficacy and improving the prognosis of patients with breast cancer (19). Studies have shown that trastuzumab, along with chemotherapeutic drugs, induces cell cycle arrest, inhibits HER2-PI3K-Akt signaling, and downregulates the expression of ERCC1 (10).

A study has shown a correlation between the expression of a few tumor markers and immune-related genes, like Programmed cell death ligand 1, as well as tumor-infiltrating lymphocytes (20). Our results show a significant negative correlation between ERCC1 expression and CD8^+^ T cells in patients with HER2 over-expressing breast cancer. Further, a correlation between high ERCC1 expression and high TIDE score was also observed, which indicates that ERCC1 expression could reduce the efficacy of immunotherapy. Furthermore, a correlation was also observed between ERCC1 expression and HRD, thus suggesting that ERCC1 expression could also reduce the efficacy of chemotherapeutic drugs like cisplatin and carboplatin. T cells play an important role in cancer, CD3 is an important T cells biomarker, and CD3 deficiency of CD3 could lead to severe combined immunodeficiency (21, 22). CD206 is an important biomarker of M2 macrophages. High CD206 levels suggest an increase in M2 macrophage infiltration, which indicates a poor prognosis for patients with breast cancer (23). IHC analysis of immune cell infiltration revealed a significantly lower T cell infiltration in patients expressing high levels of ERCC1 compared to the patients with low ERCC1 expression. Interestingly, patients with high ERCC1 expression had significantly high macrophage infiltration compared to patients with low ERCC1 expression. These results indicate that ERCC1 expression may affect the infiltration of T cells and macrophages. However, the sample size of the study is limited; few samples were available for analyzing the correlation between ERCC1 expression and T cell and macrophage infiltration. Therefore, sample collection for further analysis is currently in progress.

Clinicopathological features, such as Ki-67, histological grade, age, gender, menstrual status, tumor size, and lymph node metastasis, influence the prognosis of patients with breast cancer (24). ERCC1 is a potential tumor marker that may interact with other clinical indicators and affect the prognosis of patients. However, there is a discrepancy in our understanding of the association between ERCC1 and breast cancer (25, 26). Our study showed that the ERCC1 expression in patients with breast cancer treated with trastuzumab had no significant correlation with age at diagnosis, menopausal status, tumor size, vascular tumor thrombus, pathological type, PR, Ki-67, lymph node metastasis (P > 0.05), but significant correlations were observed among ERCC1, chemotherapy and ER repression (P < 0.05).

A correlation was observed between ERCC1 and ER expression. High ERCC1 expression was observed in patients with ER-positive breast cancer, whereas the ERCC1 expression was low in patients with triple-negative breast cancer (27). Another study showed that the expression of ERCC1 in ER-positive patients with breast cancer after neoadjuvant chemotherapy is significantly lower than that of ER-negative patients (28). We analyzed the correlation between clinicopathology features and ERCC1 expression, and the results revealed a negative correlation between ERCC1 and ER expression. Approximately 40% of patients with ER-positive breast cancer have defects in the DNA repair mechanism, which is an important factor contributing to endocrine resistance in breast cancer. A study revealed that compared to patients with low ERCC1 expression, patients with high ERCC1 expression were more likely to develop drug resistance to hormone therapy and had a poor prognosis (HR = 1.4, P = 0.02). The decrease in expression of ERCC1 and genes associated with DNA damage repair arrest the cell cycle at the G1-S phase in breast cancers, thereby altering the regulation of the cell cycle by ER. This induces resistance to hormone therapy, thereby affecting the prognosis of patients (29).

Vascular tumor thrombus is closely associated with the prognosis of patients with breast cancer. As a common pathological indicator of breast cancer, the presence of vascular tumor thrombus usually indicates invasion of endothelial lymphatic vessels and/or blood vessels by tumor cells to form emboli and release tumor cells through lymphatic and blood vessels. This increases the risk of lymph node and distant metastasis in patients with breast cancer. Vascular tumor thrombus indicates poor prognosis in patients with breast cancer and can predict distant metastasis and local recurrence of tumors (30, 31). Multivariate regression analysis revealed that vascular tumor thrombus is an independent prognostic factor for patients with breast cancer. Our results are consistent with the previous studies, which suggest that the presence of vascular tumor thrombus indicates a poor prognosis for patients with breast cancer.

ERCC1 expression also affects the chemosensitivity of the breast cancer cells. ERCC1 weakens the therapeutic effect of chemotherapy drugs by attenuating the DNA damage caused by platinum and other chemotherapy drugs and promoting DNA repair (32). In patients with triple-negative breast cancer, high expression of ERCC1 was observed in the DNA repair pathway, thereby altering the sensitivity of cancers to chemotherapeutic drugs and DNA damage inhibitors. This induces drug resistance and affects the prognosis of patients with breast cancer (33). Bioinformatic analysis revealed a correlation between ERCC1 and HRD. High-level expression of ERCC1 usually indicate the higher resistance of chemotherapy in breast cancer. No significant correlation between ERCC1 expression and TMB, as well as MSI, was observed. Our results revealed a significant correlation between chemotherapy and ERCC1 expression. Patients with high ERCC1 expression did not benefit from chemotherapy, which indicates ERCC1 alters the sensitivity to chemotherapeutic agents.

A study has revealed that increased methylation of ERCC1 in the proximal DNA in leukocytes increases the risk of breast cancer (adjusted OR = 1.29; 95% CI, 1.06-1.57) occurrence ( (34). Therefore, the ERCC1 methylation could be associated with the occurrence of breast cancer. However, due to the limited experimental samples, the relationship between the methylation status of ERCC1 and breast cancer could not be explored in this study and will be investigated by us in future studies.

Survival analysis performed on data retrieved from publicly available databases did not show a significant correlation between ERCC1 expression and patient prognosis. However, our survival analysis revealed a correlation between ERCC1 expression and patient expression. We believe that the differences in gene expression from various sources and the final results of survival analysis could contribute to discrepancies in the results. Moreover, we evaluated the correlation between ERCC1 expression and clinicopathological features, and the results revealed a correlation between ER and ERCC1 expression. However, limited information is available on the underlying mechanism or pathway by which ERCC1 affects other clinicopathological features; hence, additional studies are required to understand the role of ERCC1 further.

## Conclusion

A negative correlation was observed between ERCC1 and ER expression in patients with HER2 over-expressing breast cancer. Moreover, ERCC1 expression and vascular tumor thrombus are independent prognostic factors influencing OS in patients with HER2 over-expressing breast cancer. Besides, ERCC1 expression is also an independent factor that influences DFS. High expression of ERCC1 suggests a poor prognosis for patients with HER2 over-expressing breast cancer.

## Data availability statement

The original contributions presented in the study are included in the article/supplementary material. Further inquiries can be directed to the corresponding author.

## Author contributions

LM designed the study concept, revised the manuscript, and approved the final version of the manuscript. XL performed IHC staining, YL performed data analysis, interpreted the result, and drafted the manuscript. All authors contributed to the article and approved the submitted version.

## Funding

This study is funded by MedicalScience Research project of Health Commission of Hebei Province.

## Acknowledgments

We would like to acknowledge the TCGA GTEx TARGET network for providing data. We would also like to thank Bullet Edits for editing this manuscript.

## Conflict of interest

The authors declare that the research was conducted in the absence of any commercial or financial relationships that could be construed as a potential conflict of interest.

## Publisher’s note

All claims expressed in this article are solely those of the authors and do not necessarily represent those of their affiliated organizations, or those of the publisher, the editors and the reviewers. Any product that may be evaluated in this article, or claim that may be made by its manufacturer, is not guaranteed or endorsed by the publisher.

## References

[1] Siegel RL Miller KD Fuchs HE Jemal A. Cancer statistic. CA Cancer J Clin (2021) 71(1):7–33. doi: 10.3322/caac.21654 33433946

[2] Li Y Ma L. Nomograms predict survival of patients with lymph node-positive, luminal a breast cancer. BMC Cancer (2021) 21(1):965. doi: 10.1186/s12885-021-08642-6 34454451 PMC8401066

[3] Duffy MJ Walsh S McDermott EW Crown J. Biomarkers in breast cancer: Where are we and where are we going? Adv Clin Chem (2015) 71:1–23. doi: 10.1016/bs.acc.2015.05.001 26411409

[4] Di Fiore PP Pierce JH Kraus MH Segatto O King CR Aaronson SA. erbB-2 is a potent oncogene when overexpressed in NIH/3T3 cells. Science (1987) 237(4811):178–82. doi: 10.1126/science.2885917 2885917

[5] Li H Zhou L Ma J Zhu Y Fan J Li N. Distribution and susceptibility of ERCC1/XPF gene polymorphisms in han and uygur women with breast cancer in xinjiang, China. Cancer Med (2020) 9(24):9571–80. doi: 10.1002/cam4.3547 PMC7774751 33067872

[6] Simon GR Sharma S Cantor A Smith P Bepler G. ERCC1 expression is a predictor of survival in resected patients with non-small cell lung cancer. Chest (2005) 127(3):978–83. doi: 10.1378/chest.127.3.978 15764785

[7] Li Z Qing Y Guan W Li M Peng Y Zhang S. Predictive value of APE1, BRCA1, ERCC1 and TUBB3 expression in patients with advanced non-small cell lung cancer (NSCLC) receiving first-line platinum-paclitaxel chemotherapy. Cancer Chemother Pharmacol (2014) 74(4):777–86. doi: 10.1007/s00280-014-2562-1 25107571

[8] Chen X Wu J Lu H Huang O Shen K. Measuring β-tubulin III, bcl-2, and ERCC1 improves pathological complete remission predictive accuracy in breast cancer. Cancer Sci (2012) 103(2):262–8. doi: 10.1111/j.1349-7006.2011.02135.x 22035021

[9] Sidoni A Cartaginese F Colozza M Gori S Crinó L. ERCC1 expression in triple negative breast carcinoma: the paradox revisited. Breast Cancer Res Treat (2008) 111(3):569–70. doi: 10.1007/s10549-007-9804-4 17987380

[10] Wang YK Wang SN Li YY Wang GP Yun T Zhu CY. Methods and significance of the combined detection of HER2 gene amplification and chemosensitivity in gastric cancer. Cancer Biomark (2018) 21(2):439–47. doi: 10.3233/cbm-170671 PMC13078271 29125480

[11] Hu J Yu A Othmane B Qiu D Li H Li C. Siglec15 shapes a non-inflamed tumor microenvironment and predicts the molecular subtype in bladder cancer. Theranostics (2021) 11(7):3089–108. doi: 10.7150/thno.53649 PMC7847675 33537076

[12] Newman AM Liu CL Green MR Gentles AJ Feng W Xu Y. Robust enumeration of cell subsets from tissue expression profiles. Nat Methods (2015) 12(5):453–7. doi: 10.1038/nmeth.3337 PMC4739640 25822800

[13] Beroukhim R Mermel CH Porter D Wei G Raychaudhuri S Donovan J. The landscape of somatic copy-number alteration across human cancers. Nature (2010) 463(7283):899–905. doi: 10.1038/nature08822 20164920 PMC2826709

[14] Bonneville R Krook MA Kautto EA Miya J Wing MR Chen HZ. Landscape of microsatellite instability across 39 cancer types. JCO Precis Oncol (2017) 2017:PO.17.00073. doi: 10.1200/po.17.00073 PMC5972025 29850653

[15] Thorsson V Gibbs DL Brown SD Wolf D Bortone DS Ou Yang TH. The immune landscape of cancer. Immunity (2018) 48(4):812–30.e814. doi: 10.1016/j.immuni.2018.03.023 29628290 PMC5982584

[16] Qiu H Cao S Xu R. Cancer incidence, mortality, and burden in China: a time-trend analysis and comparison with the united states and united kingdom based on the global epidemiological data released in 2020. Cancer Commun (Lond) (2021) 41(10):1037–48. doi: 10.1002/cac2.12197 PMC8504144 34288593

[17] Callagy G Cattaneo E Daigo Y Happerfield L Bobrow LG Pharoah PD. Molecular classification of breast carcinomas using tissue microarrays. Diagn Mol Pathol (2003) 12(1):27–34. doi: 10.1097/00019606-200303000-00004 12605033

[18] Griguolo G Bottosso M Vernaci G Miglietta F Dieci MV Guarneri V. Gene-expression signatures to inform neoadjuvant treatment decision in HR+/HER2- breast cancer: Available evidence and clinical implications. Cancer Treat Rev (2022) 102:102323. doi: 10.1016/j.ctrv.2021.102323 34896969

[19] Gianni L Colleoni M Bisagni G Mansutti M Zamagni C Del Mastro L. Effects of neoadjuvant trastuzumab, pertuzumab and palbociclib on Ki67 in HER2 and ER-positive breast cancer. NPJ Breast Cancer (2022) 8(1):1. doi: 10.1038/s41523-021-00377-8 35013314 PMC8748500

[20] Gonzalez-Ericsson PI Stovgaard ES Sua LF Reisenbichler E Kos Z Carter JM. The path to a better biomarker: application of a risk management framework for the implementation of PD-L1 and TILs as immuno-oncology biomarkers in breast cancer clinical trials and daily practice. J Pathol (2020) 250(5):667–84. doi: 10.1002/path.5406 32129476

[21] de Saint Basile G Geissmann F Flori E Uring-Lambert B Soudais C Cavazzana-Calvo M. Severe combined immunodeficiency caused by deficiency in either the delta or the epsilon subunit of CD3. J Clin Invest (2004) 114(10):1512–7. doi: 10.1172/jci22588 PMC525745 15546002

[22] Yuan L Xu J Shi Y Jin Z Bao Z Yu P. CD3D is an independent prognostic factor and correlates with immune infiltration in gastric cancer. Front Oncol (2022) 12:913670. doi: 10.3389/fonc.2022.913670 35719985 PMC9198637

[23] Strack E Rolfe PA Fink AF Bankov K Schmid T Solbach C. Identification of tumor-associated macrophage subsets that are associated with breast cancer prognosis. Clin Transl Med (2020) 10(8):e239. doi: 10.1002/ctm2.239 33377644 PMC7719284

[24] Musallam R Alnajjar M Al-Shurafa A Bottcher B. Clinical and pathological characteristics and hormone receptor status of women with breast cancer in the European Gaza hospital: a retrospective chart-based review. Lancet (2021) 398 Suppl 1:S38. doi: 10.1016/s0140-6736(21)01524-5 34227971

[25] Gerhard R Carvalho A Carneiro V Bento RS Uemura G Gomes M. Clinicopathological significance of ERCC1 expression in breast cancer. Pathol Res Pract (2013) 209(6):331–6. doi: 10.1016/j.prp.2013.02.009 23702380

[26] MA ELB El Kashef WF. ERCC1 expression in metastatic triple negative breast cancer patients treated with platinum-based chemotherapy. Asian Pac J Cancer Prev (2017) 18(2):507–13. doi: 10.22034/apjcp.2017.18.2.507 PMC5454751 28345838

[27] Kim D Jung W Koo JS. The expression of ERCC1, RRM1, and BRCA1 in breast cancer according to the immunohistochemical phenotypes. J Korean Med Sci (2011) 26(3):352–9. doi: 10.3346/jkms.2011.26.3.352 PMC3051081 21394302

[28] Colleoni M Bagnardi V Rotmensz N Gelber RD Viale G Pruneri G. Increasing steroid hormone receptors expression defines breast cancer subtypes non responsive to preoperative chemotherapy. Breast Cancer Res Treat (2009) 116(2):359–69. doi: 10.1007/s10549-008-0223-y 18941889

[29] Anurag M Punturi N Hoog J Bainbridge MN Ellis MJ Haricharan S. Comprehensive profiling of DNA repair defects in breast cancer identifies a novel class of endocrine therapy resistance drivers. Clin Cancer Res (2018) 24(19):4887–99. doi: 10.1158/1078-0432.ccr-17-3702 PMC6822623 29793947

[30] Fujisawa M Omori M Doihara H Than YM Swe HWW Yoshimura T. Elastin and collagen IV double staining: A refined method to detect blood vessel invasion in breast cancer. Pathol Int (2020) 70(9):612–23. doi: 10.1111/pin.12971 32542969

[31] Huang Y Chen L Tang Z Min Y Yu W Yang G. A novel immune and stroma related prognostic marker for invasive breast cancer in tumor microenvironment: A TCGA based study. Front Endocrinol (Lausanne) (2021) 12:774244. doi: 10.3389/fendo.2021.774244 34867821 PMC8636929

[32] Li J Sun P Huang T He S Li L Xue G. Individualized chemotherapy guided by the expression of ERCC1, RRM1, TUBB3, TYMS and TOP2A genes versus classic chemotherapy in the treatment of breast cancer: A comparative effectiveness study. Oncol Lett (2021) 21(1):21. doi: 10.3892/ol.2020.12282 33240427 PMC7681196

[33] Lee KJ Mann E Wright G Piett CG Nagel ZD Gassman NR. Exploiting DNA repair defects in triple negative breast cancer to improve cell killing. Ther Adv Med Oncol (2020) 12:1758835920958354. doi: 10.1177/1758835920958354 32994807 PMC7502856

[34] Sturgeon SR Sela DA Browne EP Einson J Rani A Halabi M. Prediagnostic white blood cell DNA methylation and risk of breast cancer in the prostate lung, colorectal, and ovarian cancer screening trial (PLCO) cohort. Cancer Epidemiol Biomarkers Prev (2021) 30(8):1575–81. doi: 10.1158/1055-9965.epi-20-1717 PMC10825794 34108140

